# Co-expression network analysis of toxin-antitoxin loci in *Mycobacterium tuberculosis* reveals key modulators of cellular stress

**DOI:** 10.1038/s41598-017-06003-7

**Published:** 2017-07-19

**Authors:** Amita Gupta, Balaji Venkataraman, Madavan Vasudevan, Kiran Gopinath Bankar

**Affiliations:** 1 Department of Microbiology, University of Delhi South Campus, Benito Juarez Road, New Delhi, 110021 India; 2 Genome Informatics Research Group, Bionivid Technology Pvt Ltd, Bengaluru, 560043 India; 3 Present Address: Department of Biochemistry and Centre for Innovation in Infectious Diseases Research, Education and Training (CIIDRET), University of Delhi South Campus, New Delhi, 110021 India

## Abstract

Research on toxin-antitoxin loci (TA loci) is gaining impetus due to their ubiquitous presence in bacterial genomes and their observed roles in stress survival, persistence and drug tolerance. The present study investigates the expression profile of all the seventy-nine TA loci found in *Mycobacterium tuberculosis*. The bacterium was subjected to multiple stress conditions to identify key players of cellular stress response and elucidate a TA-coexpression network. This study provides direct experimental evidence for transcriptional activation of each of the seventy-nine TA loci following mycobacterial exposure to growth-limiting environments clearly establishing TA loci as stress-responsive modules in *M. tuberculosis*. TA locus activation was found to be stress-specific with multiple loci activated in a duration-based response to a particular stress. Conditions resulting in arrest of cellular translation led to greater up-regulation of TA genes suggesting that TA loci have a primary role in arresting translation in the cell. Our study identifed *higBA2* and *vapBC46* as key loci that were activated in all the conditions tested. Besides, *relBE1*, *higBA3*, *vapBC35*, *vapBC22* and *higBA1* were also upregulated in multpile stresses. Certain TA modules exhibited co-activation across multiple conditions suggestive of a common regulatory mechanism.

## Introduction

*Mycobacterium tuberculosis*, the causative pathogen of the deadly disease tuberculosis, has evolved mechanisms to persist in the human host for long times in a non-replicating and drug-tolerant state, and reactivate at a later time to cause disease. The underlying mechanisms and cellular factors that govern the transition of the bacterium from an active state to latent state are poorly understood^1,2^.

Recent studies in *Escherichia coli* and other bacteria point out a prominent role for toxin-antitoxin (TA) systems in stress survival and bacterial persistence^3–7^. TA loci are typically two-component systems that consist of a stable toxin and a relatively unstable antidote of the toxin, the antitoxin, both being encoded as an operon^8^. In most cases, the operon is auto-regulated at the level of transcription by the antitoxin alone and in some cases by the toxin-antitoxin complex^9–11^. Degradation of the labile antitoxin serves as a mechanism for transcriptional induction of TA locus genes as well as for the availability of free toxin protein to act on its cellular target. The most common targets of TA-encoded toxins are translation components; some others have been shown to target proteins belonging to the replication machinery^12–19^. TA-encoded toxins act to rapidly slow down cellular processes, giving bacteria an opportunity to alter their metabolic program and enter into a dormant or non-replicating state and exhibit a persistent or drug-tolerant phenotype^4,6,7,20–22^. Cells unable to adapt to this metabolic slow down initiate a programmed cascade of events leading to cell death^23–25^.

Thirty-eight TA loci were initially reported in *M. tuberculosis*^26^, however, more are being identified^27^. Presently, *M. tuberculosis* is reported to have 79 TA loci spanning the entire genome^28^. Several reports have been published on the characterization of these loci, identification of the target sites for toxins, structural characterization of some antitoxins, evaluation of their killing effect in *E. coli* and expression under stress^27,29–39^. Stochastic induction of specific TA systems has been proposed to play a role in persistence of *M. tuberculosis* in the host^40–42^.

Considering the fact that *M. tuberculosis* harbors an unusually high number of TA loci for any bacterial genome and more importantly, significantly higher than other closely related mycobacteria like *M. avium*, *M. bovis* and *M. smegmatis*, it will be useful to analyze the expression profiles of TA genes under different stress conditions thought to mimic the host environment during mycobacterial infection to elucidate their contribution towards the survival ability of this deadly pathogen. While ectopic expression of certain toxins has been shown to inhibit growth of *M. tuberculosis in vitro* and certain TA loci get induced under specific stress^31,32,35,43^, a comprehensive profile of genome-wide TA loci of *M. tuberculosis* is lacking. A system-wide screening will help identify specific TA loci differentially expressed under different growth conditions and also shed light on co-regulated pathways/genes that could provide clues to mechanisms for TA activation and regulation.

We have developed a whole-genome microarray platform for *M. tuberculosis* that harbors specific probes for all the 79 TA loci^44^. Prior published microarrays for *M. tuberculosis* did not contain probes for all these 79 TA loci. In the present study, we elucidate TA expression patterns in response to chemical and environmental stress and identify key TA loci that could have potential roles in the pathogenesis of *M. tuberculosis*. This is the first study covering various stress conditions across multiple time points on a single platform with key emphasis on genome-wide TA loci of *M. tuberculosis*.

## Results

### Expression profiling of TA loci in *M. tuberculosis*

**Figure 1:**
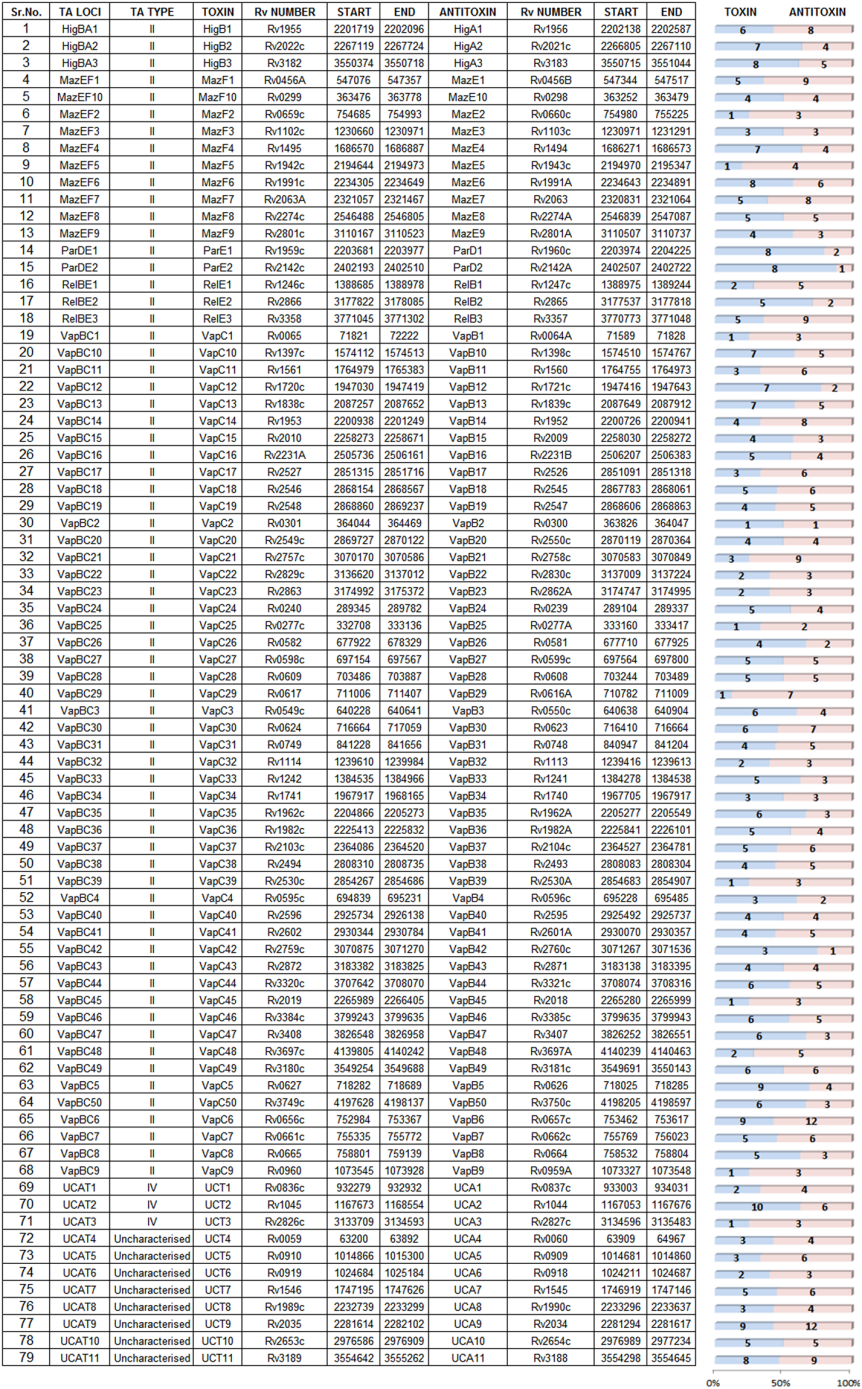
Toxin Antitoxin loci encoded by *Mycobacterium tuberculosis* H37Rv The 79 TA loci present in M. tuberculosis H37Rv genome tabulated with the locus name, gene name for toxin and antitoxin, gene identifier (Rv number), genomic location as Start base position and End base position, orientation in the genome and the type of TA locus. The uncharacterized TA loci are given the nomenclature as ucAT followed by a number for the series. The number in the blue bar indicates the total number of experimental conditions wherein differential expression was observed for the toxin gene and the number in the pink bar indicates the same for the antitoxin gene.

TA loci identified in various bacterial genomes are grouped into families based on sequence homology and biochemical properties^26^. *M. tuberculosis* H37Rv genome harbours 79 TA loci spanning the entire genome (Fig. 1) of which 68 loci have been assigned to the existing TA families. There are three *higBA* loci, ten *mazEF* loci, two *parDE* loci, three *relBE* loci, and fifty *vapBC* loci. The remaining 11 loci (*ucAT*) are uncharacterised and yet to be assigned to existing/novel TA families.

Using a recently described *M. tuberculosis* microarray covering all these 79 TA loci^44^, we obtained the expression profile of *M. tuberculosis* H37Rv after subjecting it to various stresses including chemical (drug) stress and environment stress (nutrient starvation and low pH). The four frontline drugs that comprise TB chemotherapy namely Rifampicin (RIF), Isoniazid (INH), Ethambutol (ETH) and Streptomycin (STR) were used in this study to understand changes in expression pattern of TA loci in response to drug administration to the bacteria. Low pH shift was used to mimic environment of mycobacteria in the phagosomal compartment of the host macrophage and starvation (STRV) was used to mimic the nutrient limitation encountered by the bacterium in the host. *M. tuberculosis* was subjected to these stresses and samples at multiple time points were used for expression studies. Quantitative RT-PCR was performed to check expression of selected marker genes and validate the cultures for stress exposure. These marker genes were selected based on published literature^45,46^. The fold change obtained for various genes correlated with published data for different conditions (data not shown). Some of these conditions have been used previously to define the transcriptome of *M. tuberculosis* by Boshoff *et al*.^45^. They have analysed the expression profile of M. tuberculosis H37Rv under different growth conditions in culture. However, their microarray design does not contain probes for all the 79 TA loci nor does their analysis focus on the expression of TA loci under different conditions. Our study aims to address this gap in existing knowledge by providing data and its analysis for all the 79 TA loci.

After transcriptional profiling of all the samples representing eighteen stress conditions, the data obtained was analysed for differentially expressed genes. As shown in Fig. 1, each of the 79 TA locus exhibits differential expression in *M. tuberculosis* in response to one or more stress conditions studied here, signifying the need of the cell to alter the expression of TA genes in response to stress. Further, most genes have altered expression across multiple stress conditions. However, no one gene is expressed under all the conditions tested. Even members from within the same TA family are differentially expressed under different set of conditions. This clearly suggests a stress-specific expression of TA genes. *higBA*1, *higBA*2, *higBA*3, *mazEF*1, *mazEF*6, *mazEF*7, *parD*1, *parD*2, *relBE*3, *vapBC*5, *vapBC*6, *vapBC*14 and *vapBC*21 loci had differential expression of their component genes in at least 50% of the stress conditions studied. Some of the uncharacterised TA loci, namely *ucAT*2, *ucAT*9 and *ucAT*11 were also expressed under multiple stress conditions. It should be noted that these 158 toxin and antitoxin genes out of an approximately 4000 total genes in the mycobacterial genome comprise 4% of the coding capacity of the bacterium and belong to a single stimulus-responsive system, the TA system. This accumulation and conservation of TA loci in large numbers uniformly across the genome and their induction in response to varied environment changes is clearly indicative of their role in increasing the pathogenic fitness of mycobacteria and contributing to its ability to survive in different niches in the host. This also emphasises the need for maintenance of TA loci in a bacterial genome and their ubiquitous presence across the bacterial kingdom.

### Stress-induced differential expression of TA loci in *M. tuberculosis*

**Figure 2:**
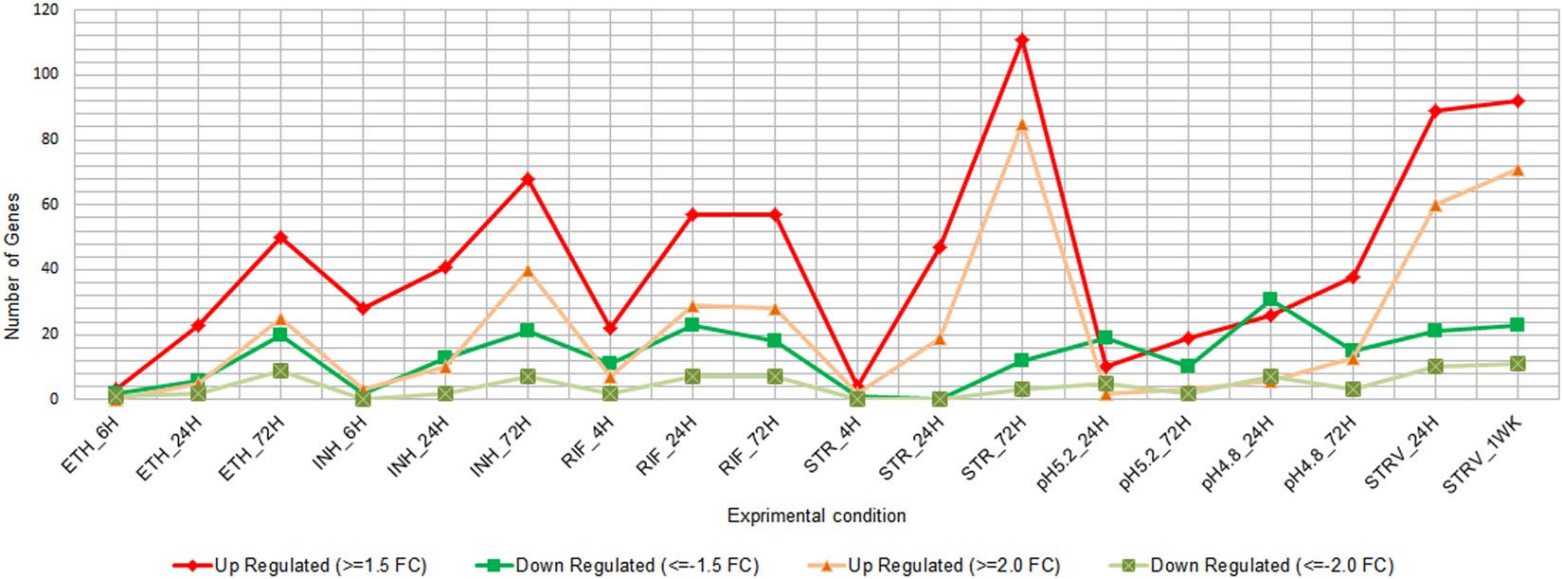
Differentially expressed TA Loci across experimental conditions. Line Graph depicts the number of TA genes that are upregulated and downregulated in each experimental condition at 1.5 and 2.0 fold change. Total number of up and down regulated toxin and antitoxin genes for each condition were plotted using Microsoft excel. The Y-axis indicates total number of toxin and antitoxin genes differentially regulated and X-axis indicates the experimental conditions and time points of the study. Ethambutol (ETH), Isoniazid (INH), Rifampicin (RIF), Streptomycin (STR), Starvation (STRV). 4 H, 6 H, 24 H, 72 H denotes the duration of treatment as 4 hours, 6 hours, 24 hours and 72 hours. 1WK denotes treatment for 1 week.

Variation in copy number of expressed transcripts leads to differences in sensitivity of detection in a microarray platform. In order to comprehensively delineate the differentially expressed TA genes in a sensitive manner, we analyzed the data with variable Fold Change (FC) cut-off at 5% confidence level. The total number of differentially expressed TA genes increased with the duration of the stress for each of the conditions (Fig. 2), suggestive of their essential and growing requirement in stress response. The greatest up-regulation of TA genes was observed in response to STR and nutrient starvation followed by RIF and INH treatments. Shift to low pH resulted in more downregulation of TA genes than upregulation. The number of differentially expressed TA genes reduced when the cutoff was raised from 1.5 to 2.0, however, greater than 50% TA genes were still differentially expressed (Supplementary Table S1). The expression pattern at 1.5 FC and 2.0 FC followed a similar trend (Fig. 2), indicating sensitivity of detection of the microarray platform. Moreover, though the number of genes was higher at lower Fold Change as expected, the TA genes identified were truly differentially expressed. The microarray data were validated using qRT-PCR for some of the TA loci. A strong positive correlation (Pearson’s correlation coefficient, R = 0.897) was observed between the microarray and qRT-PCR data (Supplementary Fig. S1).

**Figure 3:**
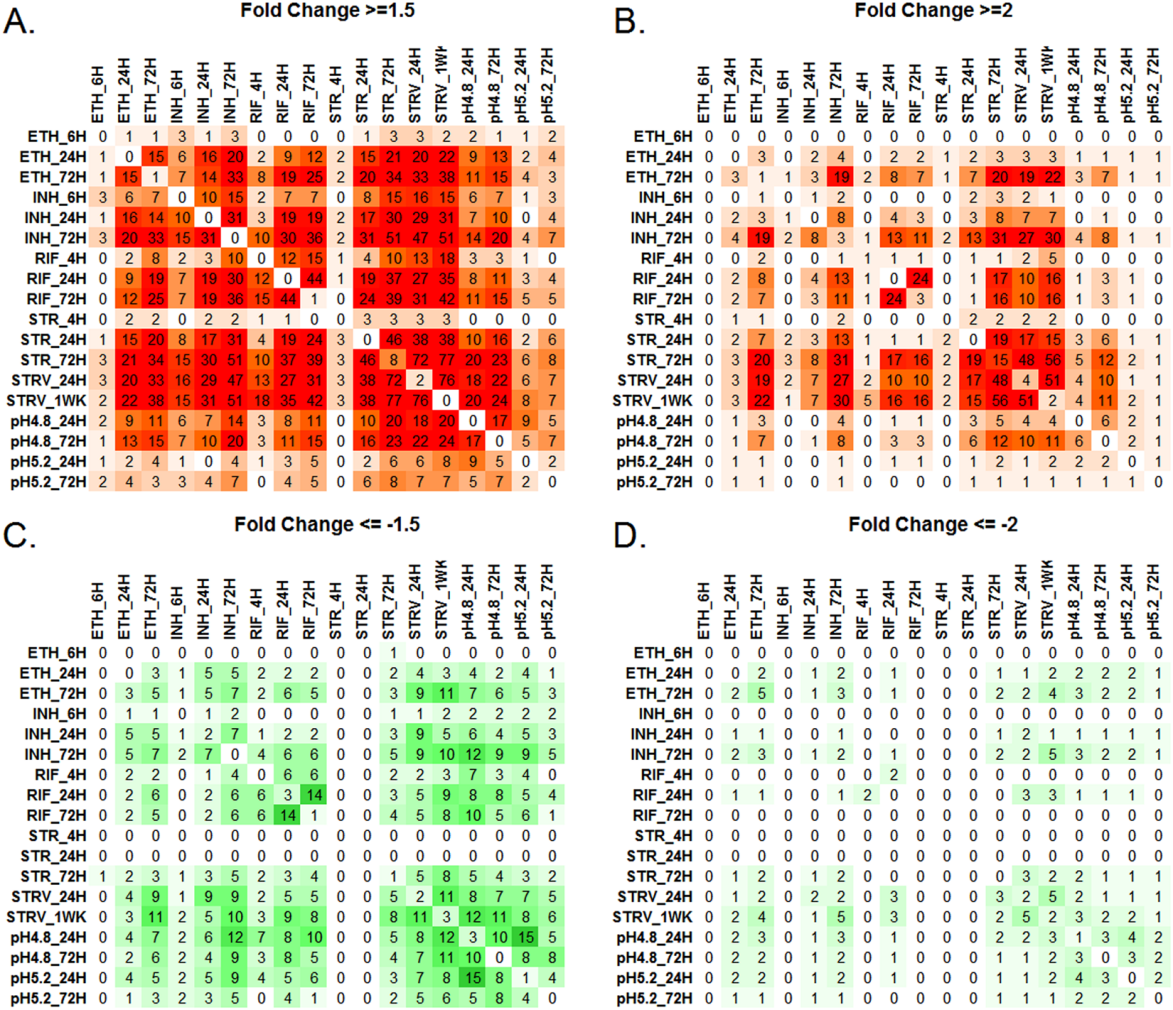
Differential gene expression matrix showing the number of up and down regulated TA genes across the experimental conditions. TA genes up and down regulated at varying sensitivity of detection were subjected to GeneMatrix software which resulted in distribution of up and down regulated genes across 18 conditions profiled. The upregulated genes at 1.5 FC (**A**) and 2.0 FC (**B**) are shown in increasing color of red while the downregulated genes at 1.5 FC (**C**) and 2.0 FC (**D**) are shown in green.

Differentially expressed TA genes unique to a given condition and those expressed across multiple conditions were identified using the matrix shown in Fig. 3. An interactive matrix showing the differentially expressed gene list for each condition is given in Supplementary Fig. S2. At 1.5 FC, 8 TA genes (*vapC*2, *vapC*32, *mazE*4, *mazE*5, *mazE*7, *vapB*38, *vapC*38 and *vapB*19) were uniquely upregulated in response to exposure to Streptomycin for 72 hours (Fig. 3A). These were not common with any other condition tested. Similarly, *vapB*18 was specifically upregulated in response to exposure to Ethambutol for 72 hours and UCT7 in response to Rifampicin for 72 hours. Two TA genes, *vapB*44 and *ucA*5, were specifically upregulated in response to nutrient starvation for 24 hours time period. 50% TA genes were common between Streptomycin and starvation stress, indicating that both the conditions induced similar TA responses in the cell. Several TA genes were found to be common under different stress conditions. Significantly higher numbers of TA genes were upregulated than downregulated under any given condition, supporting the role of TA loci as stress managers in the cell. At 2.0 FC (Fig. 3B and D), more TA genes unique to a given condition were identified. Here also, up-regulation of TA genes in response to streptomycin and nutrient starvation was maximal.

Analysis of the matrix suggests that TA gene transcription is under tight regulation. A marginal change in sensitivity of copy-number of detection by increase in FC from 1.5 to 2.0 leads to a significant drop in the number of differentially expressed TA genes, indicating a likely stringent regulatory control on gene expression. At the same time, a reduction in FC does not substantially increase the number of condition-specific genes, validating that genes identified specific to a given condition are truly specific.

**Figure 4:**
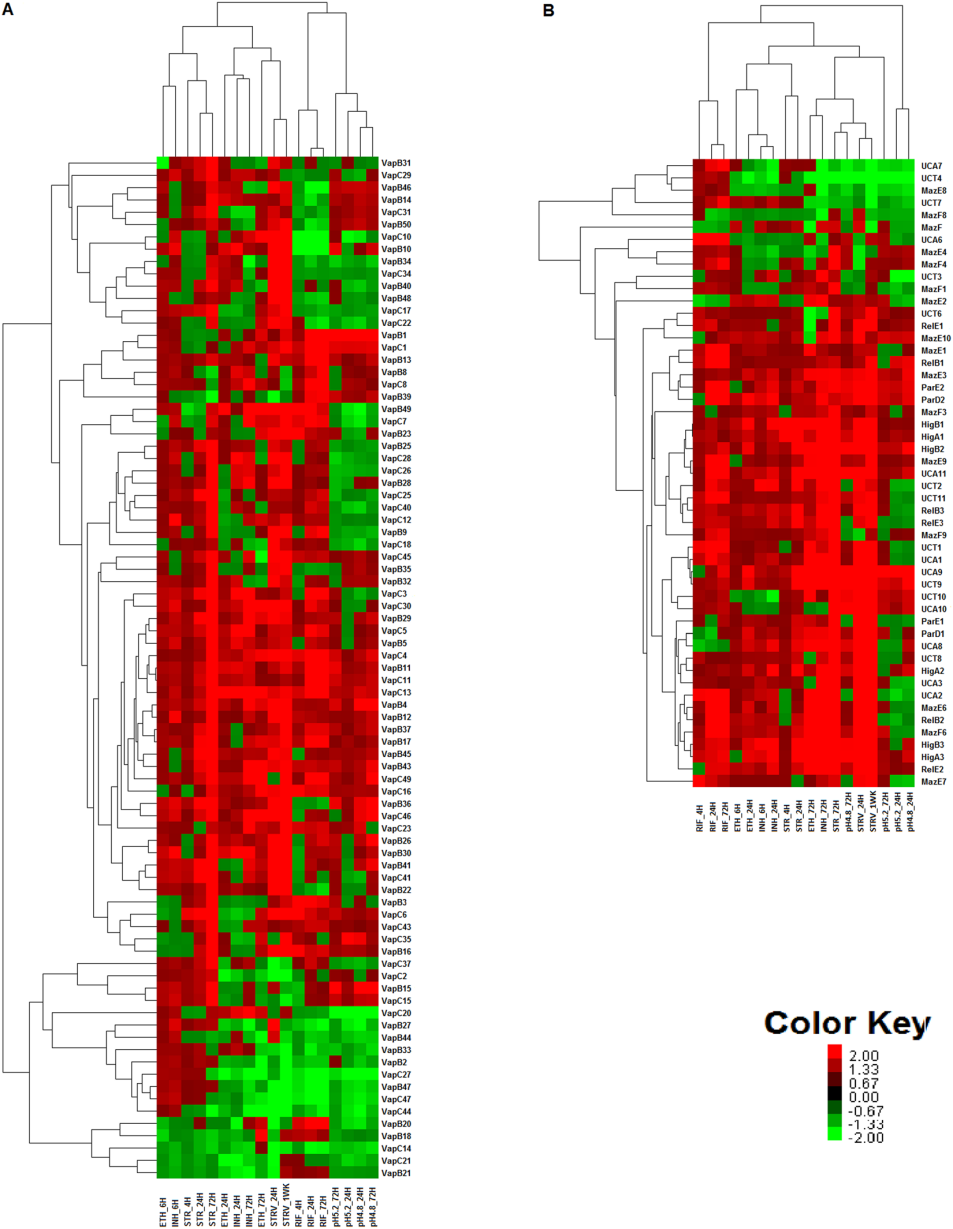
Unsupervised hierarchical clustering analysis of Differentially expressed TA Loci TA genes 1.5 fold up and down regulated along with their fold change were subjected to unsupervised hierarchical clustering. Up regulated genes are in red color gradient and down regulated genes are in green colour gradient. Fold change of 2 was given as a cutoff to render the cluster image to identify patterns of up and down regulated genes.

Unsupervised hierarchical cluster analysis of differentially expressed TA loci under different conditions was carried out (Fig. 4). Here again, the TA response clustered nutritional starvation with prolonged exposure to Streptomycin, indicating that the two stress conditions activate similar responses and pathways. The cell wall inhibitors, INH and ETH clustered together while the transcription inhibitor RIF gave a different response. Further, the 72 hours response clustered separately from the shorter duration response signifying the need of the cell to alter the expression of TA genes in response to the duration of stress. The drug–induced response clearly clustered separate from the pH stress-induced response in line with the earlier observation wherein pH was found to significantly down-regulate TA gene expression in contrast to other conditions. The cluster analysis highlights the diversity and division of labour in a biological organisation wherein even a small component like TA system gives a differential, stimulus- specific response.

### Impact of frontline TB drugs on transcription of TA loci

The four anti-TB drugs used in this study target different cell components and pathways. We performed an in-depth study of the *M. tuberculosis* transcriptome for cells treated with these drugs for three time points, to capture the primary effect of the drug as well as extended treatment response.

Treatment with Streptomycin had the most profound effect on TA expression. Streptomycin interferes with several steps of protein synthesis, its most conspicuous effect being the stimulation of translational errors and a slowing down of translocation resulting in the production of faulty proteins. A large number of TA loci were differentially regulated in response to this drug. Most loci were upregulated with increased duration of drug exposure (Fig. 4 and Supplementary Table S1). The toxin as well as antitoxin genes were upregulated for all the three *higBA* loci with progressively increasing levels. The toxin partner showed higher transcript levels compared to the cognate antitoxin. *higB*1 toxin showed a 25-fold increase, *higB*2 showed a 23-fold increase and higB3 showed a 10-fold increase in expression levels following exposure of the cell to STR for 72 hours. The toxin component of the uncharacterized TA locus *ucAT*9 showed a 21-fold increase in transcript levels. Both genes comprising *relBE*3, *vapBC*25 and *vapBC*31, *vapBC*36, *vapBC*41, *vapBC*45 and *vapBC*46 loci were also upregulated 2–10 fold at 24 hrs post-drug exposure. At 72 hrs of exposure to STR, 25 loci were upregulated. For some of these loci including *mazEF*5, *mazEF*8, *vapBC*18, *vapBC*20, *vapBC*24 and *vapBC*33, the toxin was upregulated while the antitoxin partner gene showed less or no upregulation.

Rifampicin, another frontline TB drug acts on the mycobacterial RNA polymerase leading to inhibition of transcription. The expression profile of TA loci in response to Rifampicin, across three time points is shown in Fig. 4. Rifampicin exposure induced differential expression of several TA loci. A total of 26 genes were upregulated and 22 genes were downregulated. At 4 hrs post-exposure, minimal changes in expression of TA loci were observed with *vapBC*16, *vapBC*23 and *vapBC*49 showing a 2–4 fold upregulation. However, by 24 hrs of drug exposure, a substantial number of TA genes were differentially expressed with several of them being upregulated 2–4 fold. *higBA*3, *mazEF*1, *mazEF*6, *mazEF*9, *parDE*2, *relBE*1, *relBE*2, *ucAT*1, *ucAT*2, *ucAT*10, *ucAT*11, *vapBC*1, *vapBC*3, *vapBC*4, *vapBC*8, *vapBC*11, *vapBC*13, *vapBC*16 and *vapBC*39 showed up-regulation of both partners at 24 hrs. *vapC*11, *vapC*24, and *vapC*40 toxins were preferentially upregulated compared to their antitoxin counterparts. *vapBC*10, *vapBC*14, *vapBC*22, *vapBC*27, *vapBC*44, *vapBC*47 and *vapBC*50 were downregulated 4–10 fold across all time-points. In an earlier study, Singh *et al*.^36^ have also reported significant upregulation of *relB*1 and *relB*2 antitoxins following exposure to Rifampicin. Their results corroborate with our observations.

The changes in expression profiles of TA loci in response to Ethambutol and Isoniazid, both of which target the cell wall system, were analyzed. While there was no significant change in TA gene expression at 6 hours of drug treatment, differential expression was observed 24 and 72 hours post-drug exposure. *vapC*7, *vapB*29, *vapC*30, *vapB*4, *vapC*4, *vapB*43, *vapB*49 and *vapC*49 were upregulated 2 fold in response to Ethambutol exposure for 72 hours while *vapBC*44, *vapC*45 and *vapC*47 were down regulated. While the fold change in expression levels was only 2 fold for most loci, *ucAT*9 showed a 13-fold upregulation for the antitoxin gene and a 5-fold upregulation for the toxin gene, clearly emerging as a locus responsive to ETH exposure. *mazE*6, *mazF*6 and *parD*2 were induced in response to both Ethambutol and Isoniazid. All the three *higBA* loci showed a 2–4 fold upregulation in response to Isoniazid exposure. *mazEF*2, *mazEF*6, *mazEF*9, *mazEF*10, *relBE*2, *relBE*3, *ucAT*1, *ucAT*2, *ucAT*3, *ucAT*8, *ucAT*9, *ucAT*11, *vapC*3, *vapBC*4, *vapBC*5, *vapBC*7, *vapBC*8, *vapC*13, *vapC*20, *vapC*23, *vapC*26, *vapBC*28, *vapBC*29, *vapBC*30, *vapB*34, *vapBC*41, *vapBC*43, *vapBC*45 and *vapBC*49 were also upregulated 2–5 fold in response to Isoniazid exposure. *ucA*7 and *vapC*27 were downregulated 2–4 fold in response to Isoniazid.

### Impact of environmental stress on transcription of TA loci

Nutrient starvation and acidic pH are two stressful environments encountered by mycobacteria in the host. Accordingly, the TA expression profile in response to these stress conditions was studied. We followed the Betts *et al*., method for starvation studies^47^ wherein the early exponential stage *M. tuberculosis* was re-suspended with PBS for starvation and monitored over 24 hrs and 1 week. Starvation led to generalized induction of TA loci, with a large number of TA loci upregulated at both 24 hrs and 1 week (Fig. 4 and Supplementary Table S1). Maximal induction was observed for *higBA*1, *higBA*2, *higBA*3, *mazEF*3, *mazEF*6, *mazEF*9, *parDE*1, *parDE*2, *relBE*1, *relBE*2, *relBE*3, *vapBC*4, *vapBC*9, *vapBC*10, *vapBC*12, *vapBC*16, *vapBC*17, *vapBC*22, *vapBC*23, *vapBC*26, *vapBC*28, *vapBC*30, *vapBC*32, *vapBC*34, *vapBC*35, *vapBC*41, *vapBC*45, *vapBC*46, *vapBC*49, *ucAT*1, *ucAT*2, *ucAT*3, *ucAT*8, *ucAT*9, *ucAT*10 and *ucAT*11. As many as twenty-eight loci were upregulated in response to starvation. Most of these TA loci encode translation-inhibiting RNases. Possibly, these toxins when activated in response to starvation, lead to inhibition of translation and selective degradation of mRNA to make nutrients available for survival under stress. *ucAT*9 showed a 36 fold induction after 24 hours of starvation and this increased to more than 100 fold induction after 1 week starvation. Similarly, *ucAT*1, *higBA*1, *higBA*2, and *higBA*3 showed a 10–18 fold upregulation. For *higBA*1, *higBA*2, *relBE*2, *ucAT*10, *vapBC*1, *vapBC*6, *vapBC*7, *vapBC*34, *vapBC*35, *vapBC*45 loci, the toxin was upregulated to higher levels compared to the antitoxin partner.

In contrast to starvation that led to generalized up-regulation of TA loci, low pH exposure led to down-regulation of TA genes (Fig. 4). The effect was more pronounced at pH 4.8 vis-à-vis pH 5.2. *higBA*2, *vapBC*1, *vapBC*4, *vapBC*15, *ucAT*10 and *ucAT*9 showed 2–5 fold upregulation following exposure to pH 4.8 for 72 hours. *mazEF*8, *ucAT*2, *ucAT*3, *ucAT*4, *ucAT*7, *vapC*7, *vapC*9, *vapC*20, *vapC*21, *vapBC*27, *vapBC*47 and *vapB*49, showed 2-fold down regulation at both pH 5.2 and pH 4.8.

### TA regulatory network under stress

**Figure 5:**
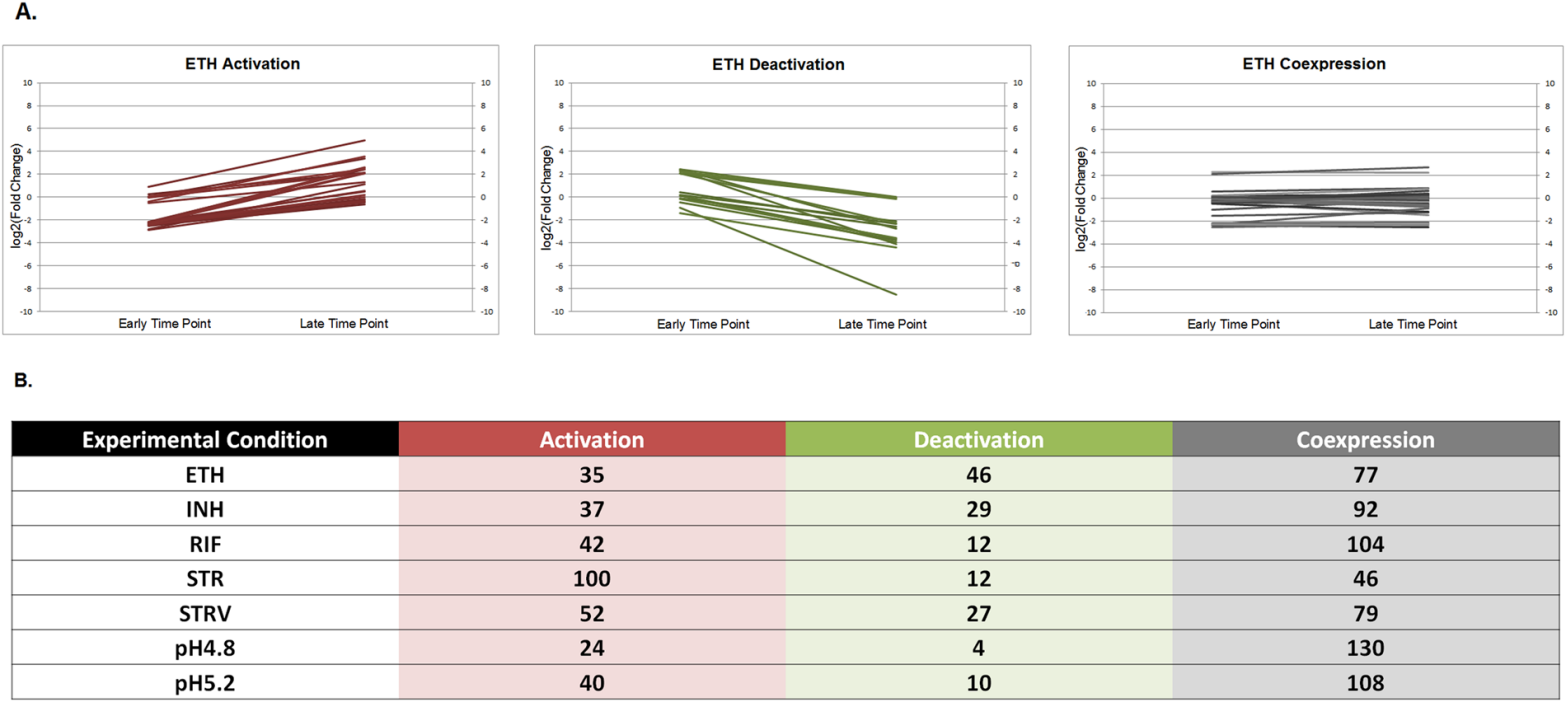
(**A**) Line Graph depicting the approach used to calculate activation and deactivation scores for TA loci (**B**) Number of TA loci that are activated, deactivated and co-expressed in each of the experimental conditions profiled. Fold change of toxin from cognate antitoxin was subtracted in early and late time point. The subtracted scores were subjected to biclustering/K-means clustering using Pearson Uncentered algorithm in GeneSpring Gx v 12.8 to provide 3 clusters of all the 78 TA loci, with clusters representing activation, deactivation and co-expression status. Similar approach was followed for each of the experimental conditions and TA loci activated, deactivated and co-expressed in each condition were identified.

**Figure 6:**
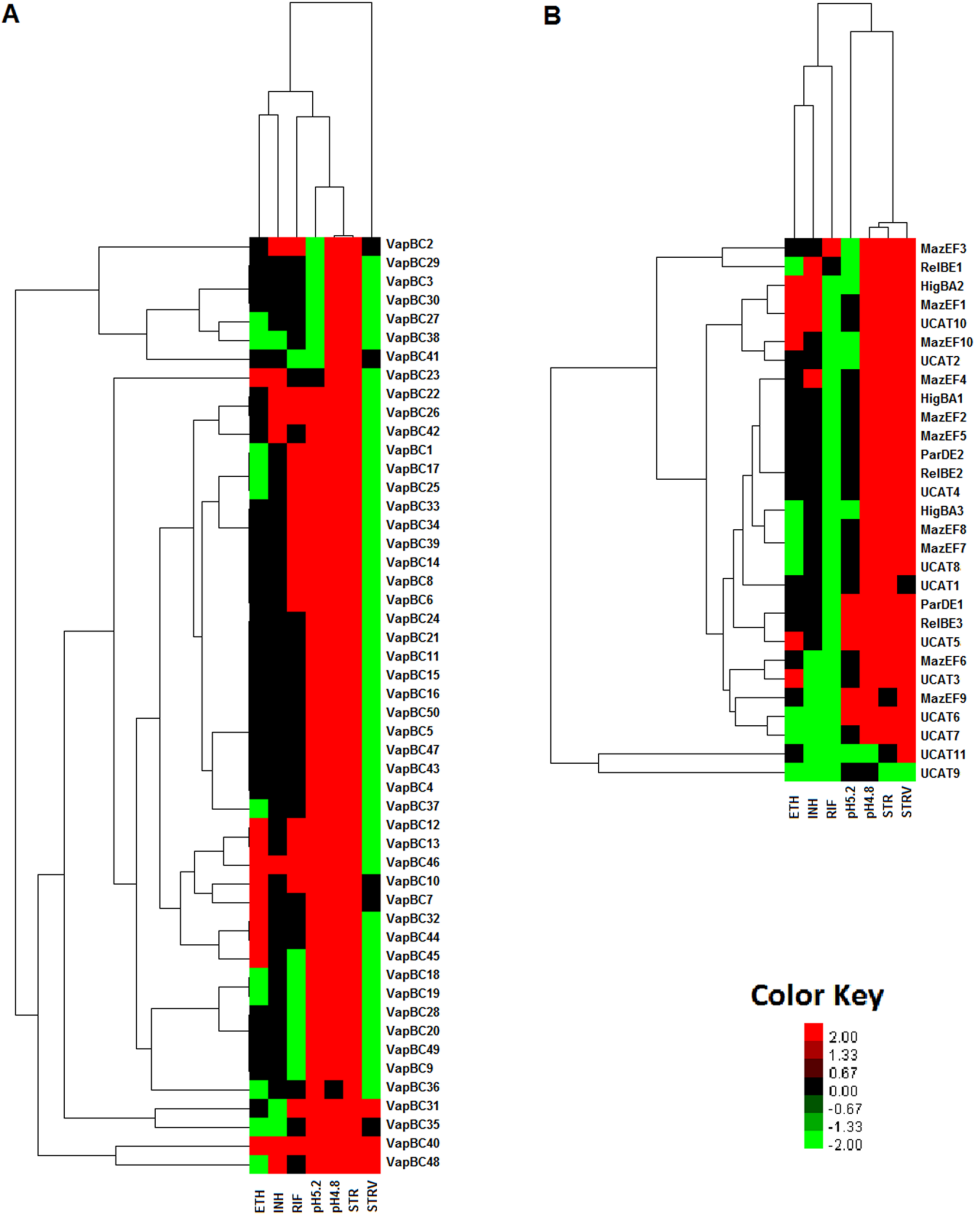
Unsupervised hierarchical clustering analysis of TA loci based on their activation and deactivation status in response to stress. All the 79 TA loci with their activation-deactivation and co-expression status defined as 2 for activation, −2 for deactivation and 0 for co-expression were subjected to unsupervised hierarchical clustering using Cluster 3.0 software by applying Pearson Uncentered algorithm with average linkage rule. The resultant cluster file was imported into Java Treeview software to visualize the cluster. Activated TA loci are in red color gradient and Deactivated TA loci are in green color gradient. A score of 2 was given as a cutoff to render the cluster image to identify patterns of activation and deactivation of TA loci across conditions profiled.

The TA transcriptome data showed that in spite of being expressed as an operon, the antitoxin and toxin levels for a given TA locus were not same across all conditions and time points (Supplementary Table S1). Based on this, we calculated the activation/deactivation scores for TA loci as described in methods to determine condition-specific TA responses. This is diagrammatically represented in Fig. 5A. If the median fold change difference between toxin transcript and antitoxin transcript level increased with duration of stress, it was designated as TA activation representing a net increase in toxin transcript vis-à-vis antitoxin transcript. The reverse was designated as TA deactivation. STR and STRV led to activation of majority of TA loci. Bi-clustering and Hierarchical clustering of the profiles based on these scores also showed a pattern of condition-based clustering (Fig. 6). The STR and STRV response was highly similar and clustered together. Both stresses led to activation of common TA loci. Nutrient starvation has been shown to cause metabolic slowdown in cells to economize on available resources, which results in arrest of translation, a phenomenon similar to Streptomycin action. The common response in the two conditions and the greater up-regulation of TA genes suggests that TA loci have a primary role in arresting translation in the cell. This is in line with the suggested mechanisms of TA loci wherein several of them are known to be mRNA interferases. The profile in response to RIF was completely in contrast to the STR and STRV response. RIF exposure led to deactivation of almost 90% TA loci. The cell wall inhibitor antibiotics, namely INH and ETH clustered separately and led to activation of some common and other different TA loci. Shift to a lower pH of 5.2 did not cause substantial changes in TA loci regulation; however, shift to pH 4.8 led to a dramatic increase in the number of activated TA loci giving a response similar to STR and STRV response. This suggests that the TA activation-deactivation response is stress-related. Different chemical and environment stress trigger varied responses in the cell and lead to activation of specific sets of TA loci, which might be helping bacteria overcome the stress.

Cluster analysis further showed that certain TA loci were co-activated under different stress conditions. *higBA1, mazEF2, mazEF5, parDE2*, *relBE2* and *ucAT4* were always activated and deactivated under same conditions indicating that they had common regulatory mechanisms. Similarly, *mazEF7, mazEF8* and *ucAT8* were co-regulated and clustered together. ucAT9 and ucAT11 showed a completely different pattern of regulation and formed a separate cluster from the other loci. Perhaps different sets of transcription factors responded to different stimuli and regulated different groups of TA loci.

**Figure 7:**
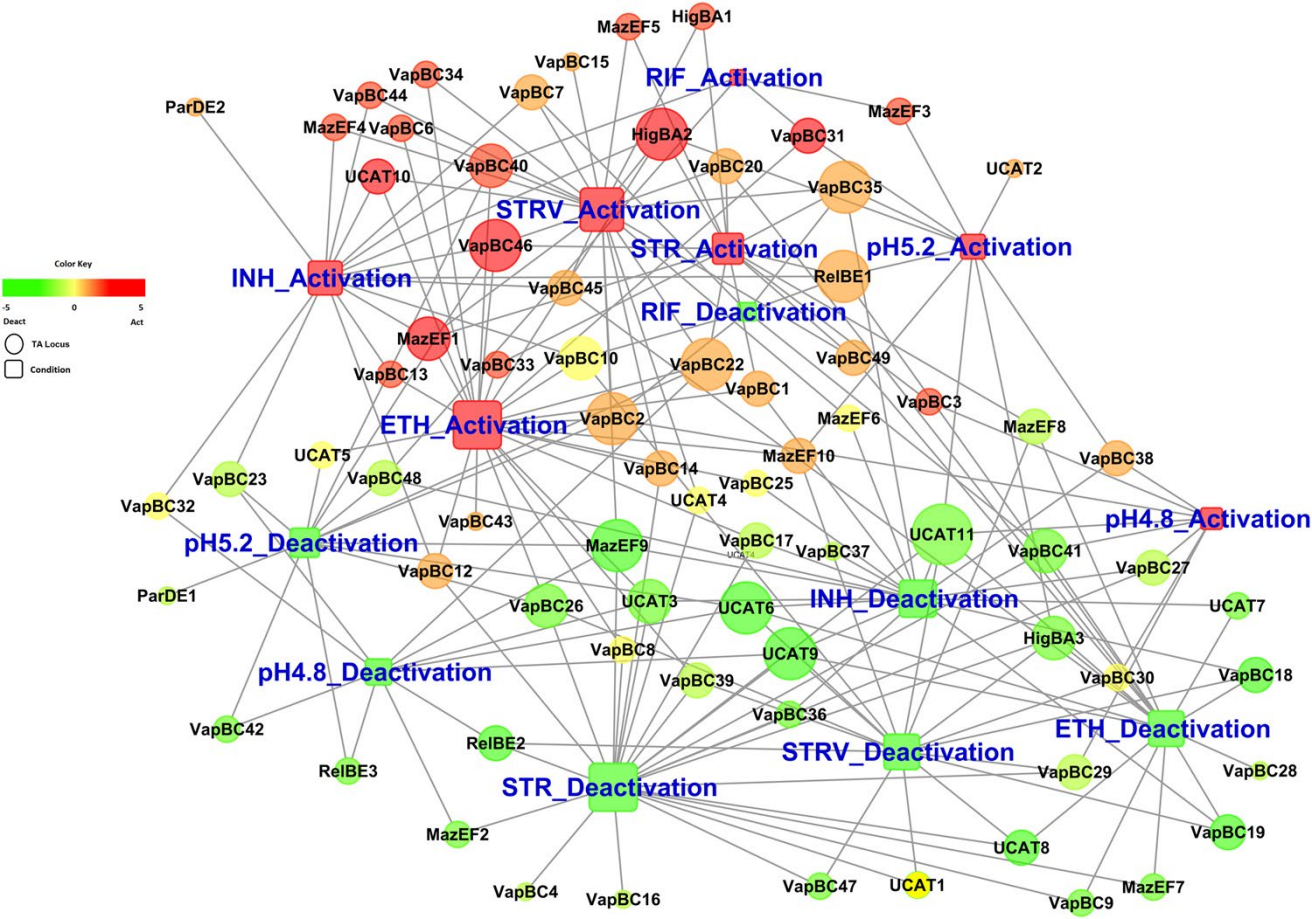
Biological Network of TA loci. Condition-based list of TA loci activated and deactivated was provided to BridgeIsland software. This software identifies the nodes and edges that come together to form a network of connections that could be representative of the TA gene regulation. The nodes (TA loci) are colored based on the activation and deactivation levels with activation being colored in red gradient and deactivation being colored in green gradient and co-expression colored in yellow gradient. The nodes are sized based on their connectivity scores with larger the size indicating a TA is activated/deactivated in as many conditions profiled.

The data were further plotted to create a TA activation-deactivation network (Fig. 7). All the TA loci were mapped based on their activation and deactivation scores with the intensity of the color correlating to the scores and the diameter of the circle corresponding to the number of conditions in which activation-deactivation was observed. As shown, there is a clear grouping of loci that are activated from those that are deactivated. *vapBC40*, *higBA*2 and *vapBC*46 were highly activated in all the conditions except low pH 5.2. *relBE*1, *vapBC*35, *vapBC*22, *vapBC*2 were also upregulated in 4 out of 6 stresses, however, the activation score was less than that for *higBA*2 and *vapBC*46. *mazEF*9 was activated in 2 conditions and deactivated in 4 conditions. ucAT11 was deactivated in all the 6 conditions studied. Other loci including *vapBC*41, ucAT6, ucAT9 and *higBA*3 were downregulated in 5 out of the 6 conditions. *vapBC*13 was uniquely activated in response to INH and ETH treatment while *parDE*2 was activated under INH stress. Several other loci were activated or deactivated under one or more conditions tested. Most loci exhibited an activation or deactivation trend across all conditions. Only 9 out of the 79 TA loci showed a mixed trend with activation under some conditions and deactivation in other conditions.

The TA activation-deactivation network clearly points out a stress-responsive activation of specific TA loci and a division of labour amongst the large number of loci. Not all TA loci are activated in response to a stress and every stress does not activate the same set of TA loci. While the cellular functions are not understood for a greater number of these loci, this data clearly indicates that the large number of TA loci maintained in mycobacterial genome does not simply comprise of pseudo genes or redundancy in gene function. Rather, these loci encode functional proteins expressed in response to specific stimulus.

### Expression of cellular proteases and nucleases under stress

**Figure 8:**
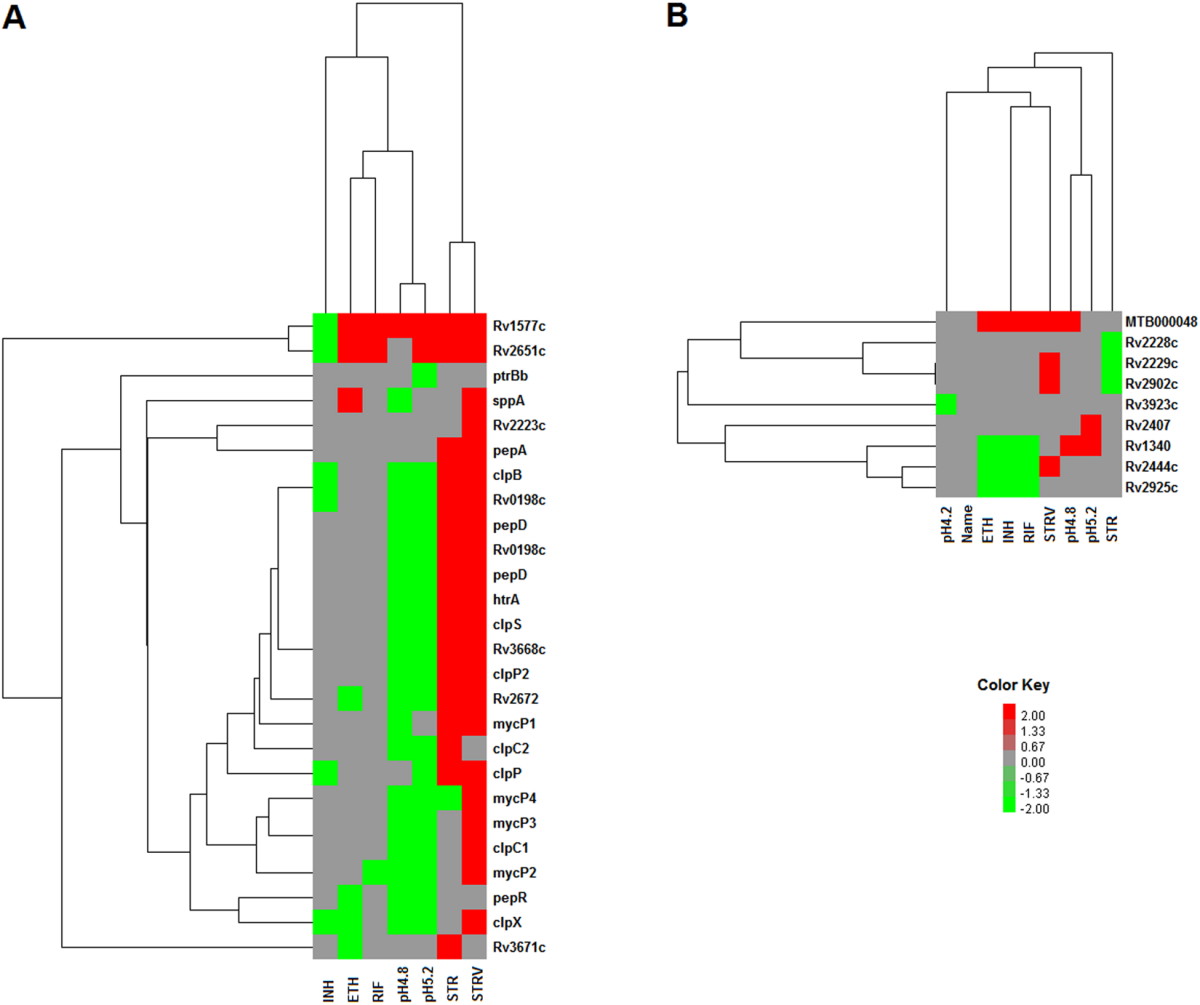
(**A**) Unsupervised hierarchical clustering analysis of protease genes based on their activation and deactivation status in response to stress. (**B**) Unsupervised hierarchical clustering analysis of nuclease genes based on their activation and deactivation status in response to stress Fold change of genes in late time point was subtracted from early time point to calculate the activation deactivation score. The cluster was created as described for Figs 5 and 6.

According to the existing model, TA loci are activated during stress by degradation of antitoxin partner by cellular proteases enabling the free toxin to exert its effect^48–51^. In this context, we checked the expression pattern of 26 annotated mycobacterial proteases under stress. As shown in Fig. 8A, several proteases were upregulated during STR and STRV stress. The Clp family proteases that have been shown to cause antitoxin degradation in several bacteria^48,50^ were found to be upregulated in our study indicating that they may play similar roles in *Mycobacterium tuberculosis*. The protease activation pattern aptly correlates with the TA activation data wherein, many TA loci were activated during STR and STRV. Two potential proteases, Rv1577c and Rv2651c were found to be upregulated under all stress conditions except INH stress. Six of the nine putative RNases of *M. tuberculosis* were also activated under one or more stress conditions (Fig. 8B). Again, maximum activation was observed under STRV and low pH stress. Further studies on these gene products can elucidate their possible roles in TA activation during stress in mycobacteria.

## Discussion

This paper is the first report providing experimental data on the expression profiles of all the seventy-nine chromosomally encoded toxin-antitoxin loci in *M. tuberculosis*. The data generated herein is used to understand activation and deactivation of TA loci in a time-dependent manner in response to environmental and chemical stresses and identify key players in cellular stress.

TA loci are being identified in many genomes and in greater numbers in each genome. This definitely raises curiosity about their possible roles in bacterial survival and persistence. *M. tuberculosis* supersedes most other bacteria for the number of TA loci it harbors in its genome. Consequently, there has been a continuing effort investigating TA loci in *M. tuberculosis*. Previous work from our lab and other groups has demonstrated killing activity of mycobacterial TA loci in heterologous hosts and native system^27,30,31,35,39,43,52–57^. Further, it has been shown that TA locus deletion strains of mycobacteria exhibit growth defective phenotype^58^.

To understand the conditions under which TA loci get induced in *M. tuberculosis*, we performed a genome-wide study and identified some key TA loci that are activated in response to chemical and/or environmental stress in *M. tuberculosis*. It is important to note here that all the 79 TA loci encoding 158 proteins in this study show specific differential regulation in one or more stress conditions. No random selection of 158 genes for any genome is expected to show such behavior supporting the conclusion that TA genes are stress-responsive and are helping bacteria tide over unfavorable conditions.

Our results corroborate with previous reports on expression of TA genes in *M. tuberculosis*^31,36,59^. Individual TA loci have been studied and shown to be induced during hypoxia, nitrogen limitation, starvation and drug exposure^27,31,32^. The *vapBC* locus in *M. smegmatis* has been implicated as playing a role in regulation of metabolic flux^60^. Singh *et al*. have also shown increased survival of *relE* overexpressing cells during drug treatment^36^. Several TA loci were identified to be upregulated in *M. tuberculosis* drug-tolerant persister population^61^. While all these studies have provided knowledge on TA loci and their activity, a comprehensive analysis of all the loci on a single platform was lacking. A genome-wide analysis of TA loci in this study has filled this gap and enabled identification of TA loci responsive to varied stress conditions. *higBA*2 and *vapBC*46 emerge as key players getting upregulated to high levels in response to chemical as well as nutritional stress. *mazEF*1, *vapBC*31, *ucAT*10 also are activated under both kinds of stress though to lesser levels. *higBA*1 and *mazEF*5 get activated in response to nutritional starvation and streptomycin treatment. Several of the TA loci identified to be activated under stress, like *mazEF*1, *vapBC*31, *higBA*1 and *mazEF*5 have been shown to have killing activity across mycobacterial species and even in heterologous systems^27,30,36^. All the three *higBA* loci in *M. tuberculosis* have been studied here and *higBA2* appears to be another important locus besides *higBA1* that has been the focus of earlier studies^56^. Many of the hitherto uncharacterised TA loci, *ucAT*11, *ucAT*6, *ucAT*9 show strong deactivation in response to both chemical and nutritional stress. Although individual deletions of TA genes have not been found to impair bacterial survival in a mouse infection model^36^, it does not rule out a role for TA loci in *M. tuberculosis* infection and survival in the human host.

Inhibition of translation is a key event in stress response and development of persistence and drug-tolerant phenotype in *E. coli*. The HigB, RelE, MazF and VapC toxins from diverse bacterial species have been identified to be mRNA interferases that cleave cellular RNA at specific sequences^15,38,43,62^. Transcriptomic analyis of *E. coli* persister cells reveals over-expression of TA modules and their encoded mRNA interferases^5,6^. Antibiotic and starvation stress has also been linked with expression of mRNA interferases^3^. The transcriptional upregulation of TA loci as observed in our study suggests a similar mechanism of translation shut-down operating in mycobacteria to impart a survival advantage to the bacteria under antibiotic pressure and nutrition deficiency. Stress-induced simultaneous activation of multiple loci would enable a rapid shutdown of cellular machinery and metabolic slowdown leading to a dormant phenotype.

In light of the above observations, TA modules could be essential genes for mycobacteria. At the same time their large number in the mycobacterial genome points towards possibilities of redundancy and pseudogene behaviour. Whole genome screens using transposon libraries have been conducted in *M. tuberculosis* to identify essential genes^63,64^. The screen includes several of the TA genes; however, none of them fall in to the category of essential genes. This is not surprising since knockout of all the ten TA loci in *E. coli* had no phenotype for normal growth and survival. Nonetheless deletion of multiple toxin genes drastically reduced tolerance to antibiotics and the level of persisters^7^ suggesting that TA genes provided selective survival advantage to cells under stress. Analysis of a recent essentiality screen data substantiates this observation^65^. Here, TA loci including *higBA1, higBA2, parDE2, VapBC6, VapBC13* and *UCAT3* are listed to confer growth advantage to mycobacteria. In our study also, *higBA1*, *higBA2* and *parDE2* are key TA modules. Essentiality of a gene for survival of the organism is dictated by the environment and a gene which is non-essential under normal growth conditions may become important under adverse conditions. TA loci appear to be one such set of genes which maybe non-essential under favourable growth conditions but confer survival advantage to the bacteria under unfavourable, stress conditions as are encountered in the human host.

Another important finding that emerges from this study is that even though the TA locus is an operon and its mRNA codes for both the toxin and antitoxin partner, the actual mRNA levels for the toxin and antitoxin genes are different. This has been reported in earlier publications^32,36^. Singh *et al*. have shown higher levels of toxin RNA compared to cognate antitoxin RNA for the three RelBE loci in lungs of *M. tuberculosis*-infected mice^36^. Korch *et al*. have also shown differences in levels of toxin and antitoxin RNA for the RelBE loci in *M. tuberculosis* when grown under hypoxic and starvation conditions^32^. The differential level of toxin and antitoxin mRNA in the cell at a given time could be a mechanism for regulating the ratio of antitoxin to toxin in the cell. Differential degradation of polycistronic RNA has been shown to determine differential expression of genes within an operon in several prokaryotic systems including *E. coli*, *Salmonella typhimurium* and *Rhodobacter capsulatus*^66–72^; however, the mechanisms involved are poorly understood. Degradation has been found to be RNaseE-dependent^70^, mRNA secondary structure based^72,73^ and small regulatory RNA (sRNA) induced^74^. Differential dosage of transcripts for *kis* and *kid* of the *parD* stability system of plasmid R1 has been shown to be a consequence of limited degradation of the polycistronic *par*D mRNA^75^. This plasmid addiction system is similar to the chromosomal toxin-antitoxin system. The expression of mycobacterial nucleases and proteases is also upregulated especially under STR and STRV stress. Protease-mediated degradation of antitoxins is well studied in *E. coli* and *Streptococcus aureus*^48–51^ and has been shown to contribute to increased toxin activity with enhanced drug resistance and persistence^76^. Similar studies in *M. tuberculosis* will shed light on the relevance of these observations and the role of nucleases and proteases in TA activation.

In summary, the present study provides vital information and clues for further studies on TA members in a genome by giving (i) direct experimental evidence for functionality of a system, (ii) configuration of the system in a genome, (iii) the activeness and responsiveness of a system to stimulus, (iv) a comprehensive analysis of the entire system across multiple time points and conditions, and (v) resolution of the system to identify key players with evidence for a division of labor across the TA system.

## Methods

### Growth of *M. tuberculosis* and RNA isolation

*M. tuberculosis* H37Rv was grown in Middlebrook 7H9 (Difco, Becton-Dickinson and Co., USA) supplemented with 0.05% Tween 80 and 10% albumin-dextrose-catalase (ADC, Difco, Becton-Dickinson and Co., USA) at 37 °C and 200 rpm, till an OD_600_ of 0.4–0.5. The culture was then split for addition of Isoniazid (isonicotinylhydrazine), Streptomycin and Ethambutol dissolved in autoclaved distilled water at a final concentration of 1 µg/ml and Rifampicin dissolved in ethanol at 0.1 µg/ml. Incubation was continued after addition for different time periods and control cultures (vehicle only) were also grown for same time periods. Cell density was measured as OD_600_ at all time points and CFU assay was performed. To prepare biological replicates for RNA isolation, each culture was independently grown in duplicate. Cells were harvested after 4, 6, 24, and 72 hrs of drug treatment and RNA was isolated. For nutrient starvation, the log phase culture of *M. tuberculosis* (OD_600_ of 0.4–0.5) was pelleted, washed twice with Phosphate Buffer Saline (PBS; 50 mM phosphate buffer, pH 7.4; 150 mM NaCl) containing 0.05% Tween 80, suspended in PBS and grown for 24 hrs and 1 week in tight-capped bottles as standing culture. Untreated culture grown in 7H9 served as control. For pH stress, the log phase culture of mycobacteria was re-suspended in Middlebrook 7H9 media with pH 5.2/pH 4.8 and grown for 24 and 72 hrs at 200 rpm. Control cultures were grown in 7H9 media with pH 6.8. The cells were harvested by centrifugation at 5000 *g* for 5 minutes at 4 °C and used for RNA isolation.

RNA isolation and Quantitative RT-PCR was performed using previously described methods^77^.

### Microarray processing and data analysis

Using random nucleotide-based T7 promoter primers included in the Low Input Quick Amp WT labelling kit (Agilent Technologies, USA), 25 ng of RNA was amplified and labelled. The labelled cRNA were purified using RNeasy columns (QIAGEN Inc, USA). cRNA yields and specific activities were measured using Nanodrop 2000c spectrophotometer (Thermo Scientific, USA). Microarrays were synthesized using the proprietary non-contact SurePrint Ink-jet technology (Agilent Technologies, USA). The microarray specifications and design have been described earlier^44^. For all arrays, the Gene Expression Hybridization Kit (Agilent Technologies, USA) and recommended protocols were employed. Hybridization and slide processing were done according to the procedure described by Agilent Technologies. Microarray slides were scanned at a resolution of 5 µm using an Agilent Microarray scanner (G2565CA) with scan control software as per the manufacturer’s instructions. Raw data obtained were normalized and analyzed as described previously^44^. Probes that showed a 2-fold or higher change with a p value < 0.05 (unpaired Student t-test) were considered to be differentially expressed.

To identify differentially expressed genes that were unique to a given condition and those that were common with other conditions, a matrix was created using GeneMatrix software (Bionivid Technology Pvt Ltd, Bangalore, India). For this, the list of differentially expressed genes in each condition was provided as input to the software that computes all condition vs all condition matrix and identifies genes differentially expressed in one condition and those expressed across multiple conditions. TA genes 1.5 fold up and down regulated along with their fold change were subjected to unsupervised hierarchical clustering using Cluster 3.0 software by applying Pearson Uncentered algorithm with average linkage rule. The resultant cluster file was imported into Java Treeview software to visualize the cluster.

For determination of TA activation levels, the difference in fold change levels of toxin transcript level to antitoxin transcript level for all stress conditions and time points was calculated. If this median fold change difference increased from an early time point to a later time point under stress, it was designated as TA activation while if it decreased from an early to a later time point, it was designated as TA deactivation. If the difference remained equal to 1, it was designated co-expression. This model was used to calculate activation/deactivation scores and determine the pattern of TA response to a particular stress condition. Bi-clustering and Hierarchical clustering using Pearson uncentered algorithm with average linkage rule was used to identify the list of TA loci that were activated and deactivated. To construct the regulatory network for TA loci, the list of activated and deactivated TA loci along with the condition in which it was measured were provided as input to BridgeIsland software (Bionivid Technology Pvt Ltd, India). BridgeIsland software identifies the connecting nodes and edges from the raw input and derives the list of connections (TA– > Activation/Deactivation– > Condition) enriched in the overall experiment. Further, this information is provided as an input to CytoScape V 2.8.2 (NIGMS, NIH, USA) to visualize the network. Force-directed spring-embedded layout algorithm was applied to the network and nodes were sized based on their connectivity score with larger nodes bearing the highest score.

## Electronic supplementary material

Supplementary data

Table S1

Supplementary Information
