# Publisher Correction: Co-expression network analysis of toxin-antitoxin loci in *Mycobacterium tuberculosis* reveals key modulators of cellular stress

**DOI:** 10.1038/s41598-018-25178-1

**Published:** 2018-05-10

**Authors:** Amita Gupta, Balaji Venkataraman, Madavan Vasudevan, Kiran Gopinath Bankar

**Affiliations:** 1 Department of Microbiology, University of Delhi South Campus, Benito Juarez Road, New Delhi, 110021 India; 2 Genome Informatics Research Group, Bionivid Technology Pvt Ltd, Bengaluru, 560043 India; 3 Present Address: Department of Biochemistry and Centre for Innovation in Infectious Diseases Research, Education and Training (CIIDRET), University of Delhi South Campus, New Delhi, 110021 India

Correction to: *Scientific Reports* 10.1038/s41598-017-06003-7, published online 19 July 2017

In the original version of this Article, the Supplementary file “Supplementary Figure S2” was inadvertently omitted. This has now been corrected in the HTML version of the paper; the PDF version was correct from the time of publication.

