# Supplementary material for: Co-expression network analysis of toxin-antitoxin loci in Mycobacterium tuberculosis reveals key modulators of cellular stress
Source: Sci Rep. 2017 Jul 19;7:5868. doi: 10.1038/s41598-017-06003-7 (PMC5517426; doi:10.1038/s41598-017-06003-7)
Supplement: Supplementary file 1 — Supplementary data [file 41598_2017_6003_MOESM1_ESM.doc]

Supplementary Information

Co-expression network analysis of toxin-antitoxin loci in Mycobacterium tuberculosis reveals key modulators of cellular stress

Amita Gupta^1^*, Balaji Venkataraman^1^, Madavan Vasudevan^2^ and Kiran Gopinath Bankar^2^

^1^Department of Microbiology, University of Delhi South Campus, Benito Juarez Road, New Delhi 110021, India

^2^Genome Informatics Research Group, Bionivid Technology Pvt Ltd, Bangalore 560043, India

* Corresponding author:

Present Address: Department of Biochemistry, University of Delhi South Campus, Benito Juarez Road, New Delhi 110021

Tel: 91-11-24114172; E-mail: amitagupta@south.du.ac.in

Supplementary Fig. S1

Validation of microarray data using real time PCR.

Bars represent fold change for the stress condition A, Isoniazid 72 Hrs; B, Streptomycin 72 Hrs; C, Starvation 24 Hrs. The Y axis shows the fold change 2-ΔΔ Ct values of qRT-PCR. The X-axis shows selected genes.

D. Correlation between expression values of selective genes obtained by microarray (x-axis) and qRT-PCR (y-axis). Normalized fold change values of microarray experiment and 2-ΔΔ Ct values of qRT-PCR obtained for the same genes were analysed for Pearson’s correlation coefficient (R = 0.879).


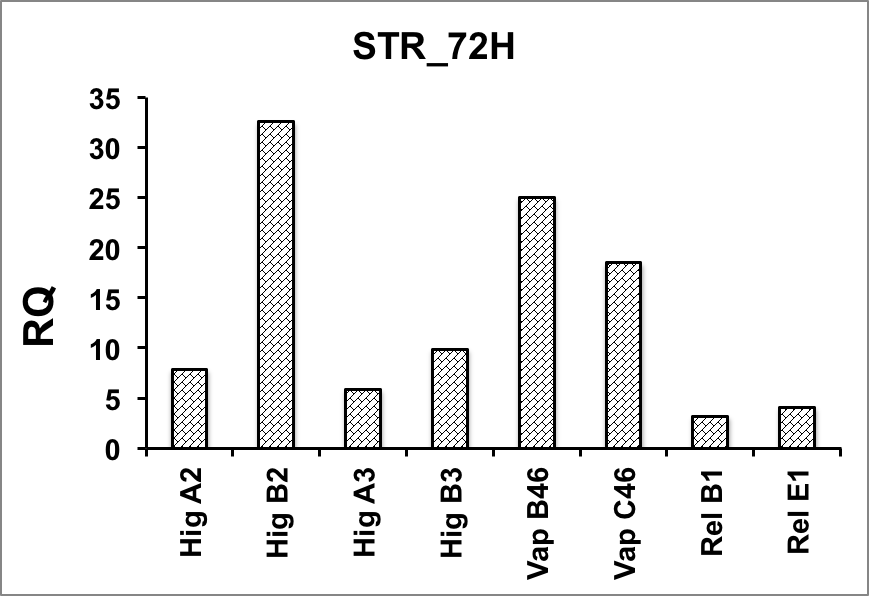


**A**


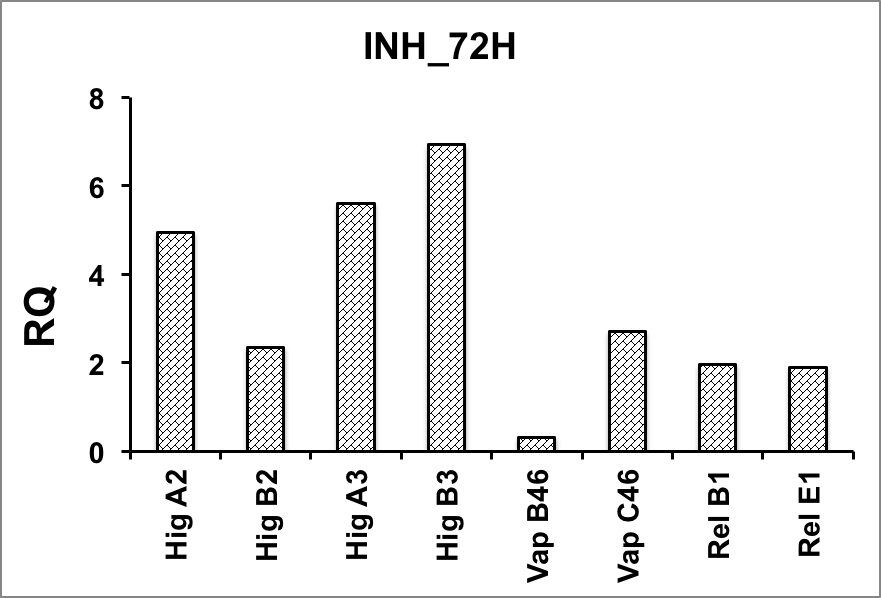


**B**

**
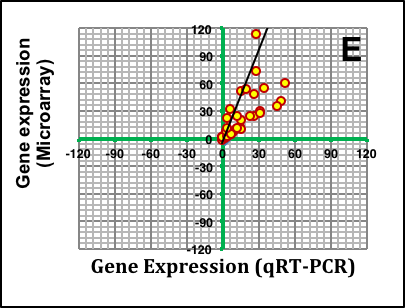

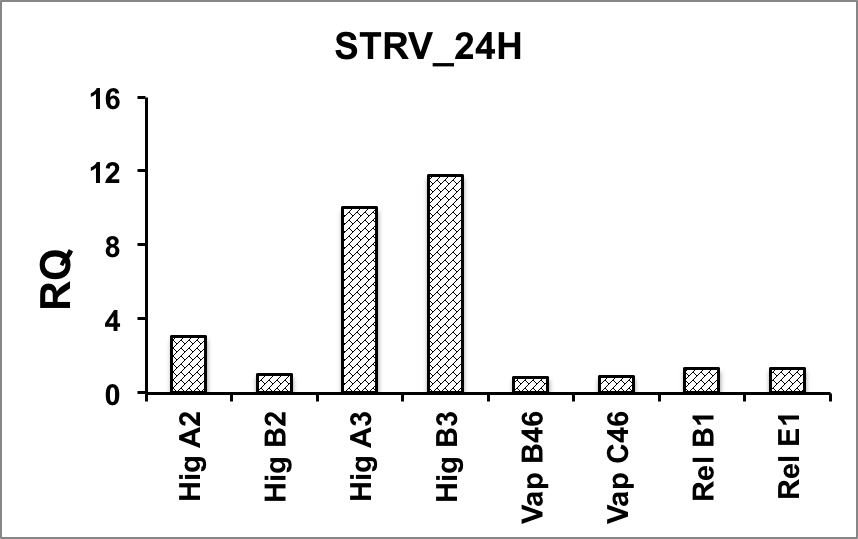
**

**C**

**D**

Supplementary Fig. S2

Interactive differential gene expression matrix showing the number of up and down regulated TA genes across the experimental conditions along with the gene list

An interactive HTML file that shows the distribution of up and down regulated TA genes across 18 conditions profiled. Moving the cursor over the number provides the list of Toxin and Antitoxin genes that are differentially expressed. The same list of genes represented by Rv number can be downloaded as MS excel file.

Supplementary Table S1

An excel sheet showing the differentially expressed TA genes across the experimental conditions at 1.5 fold change along with the probe Id for each gene and the values for differential expression. The p value for the each analysis is given in a separate spreadsheet.
