# Supplementary material for: Co-expression network analysis of toxin-antitoxin loci in Mycobacterium tuberculosis reveals key modulators of cellular stress
Source: Sci Rep. 2017 Jul 19;7:5868. doi: 10.1038/s41598-017-06003-7 (PMC5517426; doi:10.1038/s41598-017-06003-7)
Supplement: Supplementary file 3 — Supplementary Information [file 41598_2017_6003_MOESM3_ESM.html]

Highcharts Example
